# Dual Kinect v2 system can capture lower limb kinematics reasonably well in a clinical setting: concurrent validity of a dual camera markerless motion capture system in professional football players

**DOI:** 10.1136/bmjsem-2018-000441

**Published:** 2018-12-17

**Authors:** Argyro Kotsifaki, Rodney Whiteley, Clint Hansen

**Affiliations:** 1 Aspetar, Orthopaedic and Sports Medicine Hospital, Doha, Qatar; 2 Department of Neurology, University of Kiel, Kiel, Germany

**Keywords:** 3D marker based system, markerless motion capture, Kinect, kinematics

## Abstract

**Objectives:**

To determine whether a dual-camera markerless motion capture system can be used for lower limb kinematic evaluation in athletes in a preseason screening setting.

**Design:**

Descriptive laboratory study.

**Setting:**

Laboratory setting.

**Participants:**

Thirty-four (n=34) healthy athletes.

**Main outcome measures:**

Three dimensional lower limb kinematics during three functional tests: Single Leg Squat (SLS), Single Leg Jump, Modified Counter-movement Jump. The tests were simultaneously recorded using both a marker-based motion capture system and two Kinect v2 cameras using iPi Mocap Studio software.

**Results:**

Excellent agreement between systems for the flexion/extension range of motion of the shin during all tests and for the thigh abduction/adduction during SLS were seen. For peak angles, results showed excellent agreement for knee flexion. Poor correlation was seen for the rotation movements.

**Conclusions:**

This study supports the use of dual Kinect v2 configuration with the iPi software as a valid tool for assessment of sagittal and frontal plane hip and knee kinematic parameters but not axial rotation in athletes.

**Highlights**

- Markerless (multiple Kinect v2) camera kinematic analysis a relatively inexpensive clinically useful tool.
- Excellent shin range flexion-extension in all tests.
- Excellent results for peak angles regarding knee flexion.
- Poor results for rotations.

## Introduction

Precompetition medical assessment of athletes commonly includes assessment of movement quality while athletes perform standardised testing procedures. Depending on the particular sport’s performance requirements and injury patterns, different test batteries are employed in an effort to identify at-risk individuals to target for tailored interventions. The quantification of these movement assessment tests is typically performed with simple visual analysis and rating,^1^ or occasionally using video recording and later 2-dimensional analysis. Such approaches have shown limited accuracy in estimating injury likelihood, and it has been suggested that this could be attributed, in part, to the reduced objectivity of these approaches in comparison to 3-dimensional kinematic analyses.

In the context of football (soccer), commonly performed functional tests include: Single Leg Squat (SLS) assessing motion in frontal plane knee motion;^2–4^ Single Leg Jump (SLJ)^5 6^ and Counter-movement Jump (CMJ) for lower limb power estimation.^7^ Additionally, a modification of the CMJ with the athlete landing on one leg instead of two (Modified Counter Movement Jump (MCMJ)) has been recommended as being more sport-specific.^8^

Marker-based motion capture is currently considered the reference method for kinematic analyses. These approaches, however, require expensive equipment, significant operator training and analysis time as well as increased subject set-up time. Accordingly, these approaches are rarely employed in settings where time and/or financial constraints exist such as preseason screening of athletes performing functional movement. Additionally, these somewhat artificial laboratory conditions can cause unknown experimental artefacts.^9^

Recent advances and improved access to markerless motion capture technology have made the use of low-cost motion analysis tools a possibility in the clinical setting.^10^ However, the validity of this technology in more complex functional movements is currently unclear. The majority of studies done so far used Kinect v1, one camera and the Software Development Kit (SDK) provided by Microsoft. Researchers have evaluated the configuration during working activities,^11^ functional activities,^12^ gait in healthy population,^13 14^ gait in multiple-sclerosis,^15^ and after cerebrovascular accident,^16^ and during a jump test.^17^ More recently, single Kinect v2 was used with Microsoft SDK to test the validity during gait^10^ and for balance.^18^ A multi-Kinect v2 configuration with Microsoft SDK was tested for its validity during gait.^19 20^ To our knowledge, until now, no validation of a dual-camera markerless system during dynamic, advanced movements has been done.

The goals of this study were to examine the validity of a markerless motion capture system using 2 Kinect v2 cameras with custom software during functional movements commonly performed during pre-season physical screening evaluation.

## Method

### Participants

Thirty-four pain-free male professional football players participated in the study(table 1). All athletes had no previous lower extremity surgery and no current injury. We followed Fleiss’ recommendation^21^ for reliability studies after considering previous work in the area.^22 23^ This study was approved by the ethical review board (Institutional Review Board E2013000003) and all participants provided written informed consent as required by the Helsinki declaration.

**Table 1:** Participant information

Participants	Mean±SD
	Male (n=34)
Age (years)	26.63 (±4.23)
Weight (kg)	73.58 (±11.44)
Height (cm)	176.01 (±8.01)
BMI (kg/m^2^)	23.62 (±2.25)

BMI, body mass index.

### Materials

**Figure 1:**
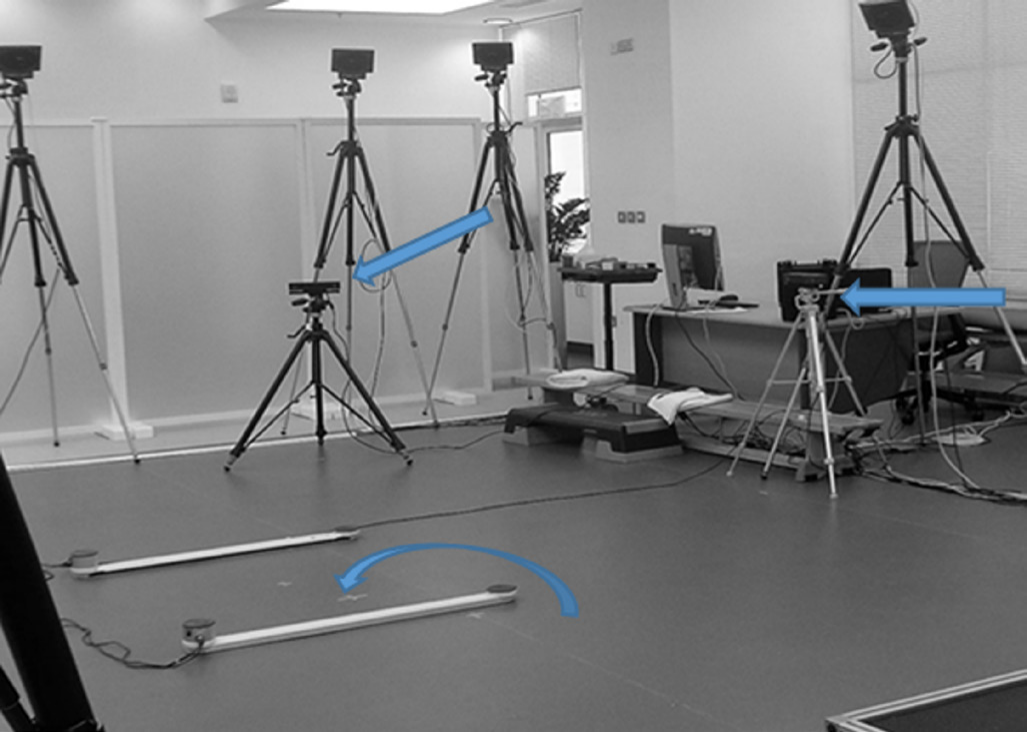
Biomechanics lab setup with marker based motion capture system and dual Kinect V2 configuration. Kinect camera placement indicated by the two straight arrows, and athletes were tested within the area of the Opto-Jump sensors (curved arrow). The athletes were tested such that they were facing one Kinect camera, with the other one to their left.

Marker trajectories were measured with a 13-camera motion capture system (BTS-SMART 1000, BTS S.p.A., Italy) sampling at 250 Hz. Depth and colour image data were simultaneously recorded with 2 Kinect v2 cameras at 30 Hz (Kinect for Windows, Microsoft, Redmond, Washington, USA) and iPi Recorder (iPi Soft, Moscow, Russia). Kinect cameras were placed one in front and one to the left side of the capture area (in between the 2 Optojump sensors) at an angle of 70° between them (figure 1).

### Data collection

**Figure 2:**
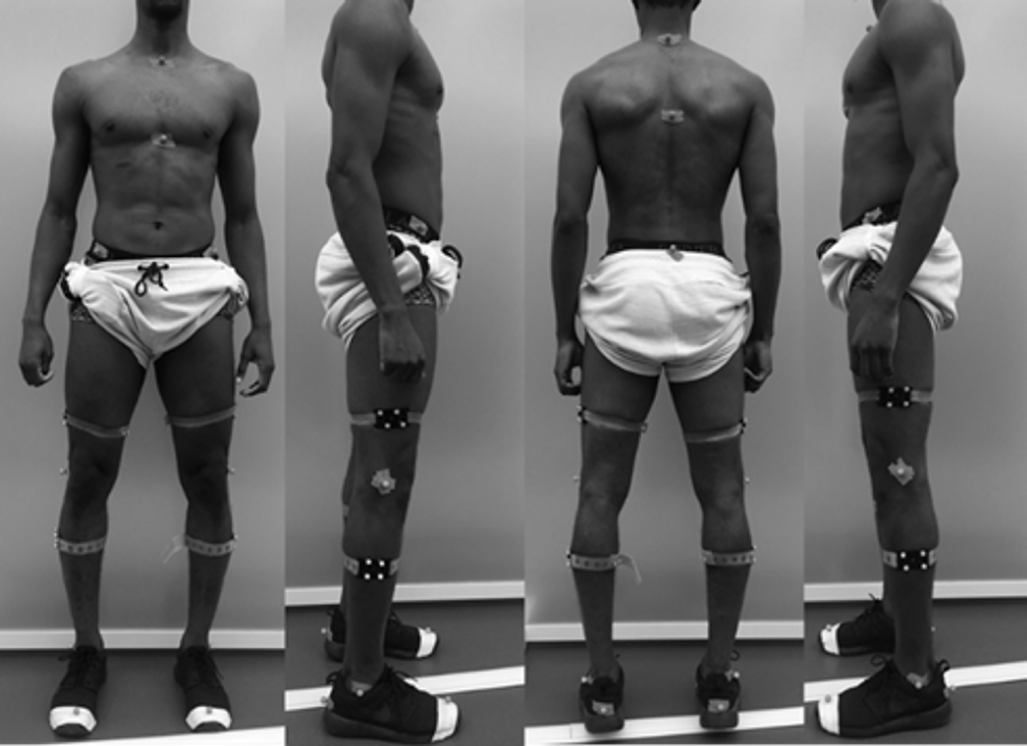
Marker placement.

After warm up for a minimum of 5 min, 31 markers were placed using clusters for thigh and shin and on anatomical landmarks according to standard marker protocol (figure 2). Participants stood in the capture area and performed three repetitions each of a SLS, a SLJ and a MCMJ, in the same order. Each trial was captured from BTS and Kinect cameras simultaneously.

### Data analysis

Kinematic data from the Kinect cameras were processed using biomechanics add-on software (iPi Soft, Moscow, Russia). Marker trajectories from the marker-based system were processed using the SMART Analyser application (BTS S.p.A., Italy). For this analysis, the trajectories were adjusted to iPi Software such that comparison of the extracted data could be made. Marker based data were filtered using Butterworth Low Pass Filter at 6 Hz and resampled at 30 Hz. Kinematic data from both systems were extracted in Euler angles (rotation sequence XYZ), in degrees, relative to the ground for thigh, shin and foot. For each trial, time synchronisation was performed manually by identifying the starting point of each trial as the moment of heel raise from the floor and the end point as the moment of heel contact to the floor.

The range of movement at the thigh, shin and foot and peak angles at the thigh and shin were averaged across three cycles in each exercise and used for subsequent analysis. Range of movement was calculated for each joint of interest as the difference between maximum and minimum angles for each cycle. Mean subject-based values for each test were then determined. Note that these were calculated independently for both the markerless (Kinect) and marker-based (BTS) equipment.

### Statistical analysis

A two-way mixed analysis of variance (ANOVA) (absolute agreement) was performed to assess the reliability and the variability of the measurements. Between measurement agreement was assessed using intraclass correlation coefficients (ICC) (2, k; absolute agreement). Because the ICC does not allow us to fully appreciate the magnitude of within-subject variance, we also calculated the SE of measurement (SEM) and the minimal detectable change (MDC).^24^ SEM represents the within-subject reliability of the measure and, consequently, the reliability of the measure.^24^ The SEM was determined as √MSE, where MSE=mean square error from the ANOVA table. The MDC represents the threshold over which an individual change can be considered meaningful when taking into account the variability associated with both the measurement technique and the experimental sample and was calculated using the equation MDC=1.96 × √2×SEM Finally, to better understand system agreement of the peak joint angles, the 95% limits of agreement and the bias were calculated using Bland-Altman analysis.^25^ The bias represents the average difference in peak joint angle between the systems while the limits of agreement are the bias ±SD. Significance level was set at p<0.05. Correlation coefficients were interpreted as follows: less than 0.40 as poor, between 0.40 and 0.59 as fair, between 0.60 and 0.74 as good, between 0.75 and 1.00 as excellent.^26^ All analyses were performed using SPSS 23.0 (SPSS Statistics for Windows, V.23.0. Armonk, New York, USA: IBM).

## Results

Mean(±SD), absolute agreement ICC, SEM and MDC values for angles and ranges of motion are provided in tables 2–4.

**Table 2:** Range of angles, averaged over the three cycles during the Single Leg Squat test for BTS (considered as reference standard) and IPI software-Kinect configuration

Test	Segment	Movement	System	Mean (SD)	95% CI		ICC(2,k) (95% CI)	P value	SEM	MDC
				(deg)	Lower (deg)	Upper (deg)			(deg)	(deg)
SLS_L (n=34)	THIGH	Flexion/Extension	BTS	42.5 (6.5)	40.2	44.7	0.532 (–0.21 to 0.84)	0.000	3.8	10.5
			iPi	52.6 (7.8)	49.8	55.3				
		Rotation	BTS	15.1 (3.2)	14.0	16.2	0.312 (–0.31 to 0.65)	0.069	3.5	9.6
			iPi	13.5 (4.5)	12.0	15.1				
		Abduction/Adduction	BTS	13.2 (4.7)	11.6	14.9	0.775 (0.55 to 0.89)	0.791	3.4	9.5
			iPi	13.5 (6.4)	11.2	15.7				
	SHIN	Flexion/Extension	BTS	26.3 (5.3)	24.4	28.1	0.886 (0.73 to 0.95)	0.006	2.4	6.7
			iPi	28.0 (6.2)	25.8	30.2				
		Rotation	BTS	21.0 (4.4)	19.5	22.5	0.126 (–0.31 to 0.47)	0.000	4.7	12.9
			iPi	15.5 (5.4)	13.6	17.4				
		Abduction/Adduction	BTS	15.5 (6.7)	13.1	17.8	0.718 (0.17 to 0.88)	0.000	3.4	9.4
			iPi	11.5 (5.2)	9.7	13.3				
	FOOT	Flexion/Extension	BTS	4.7 (4.0)	3.3	6.0	0.324 (–0.20 to 0.68)	0.000	3.1	8.6
			iPi	12.2 (4.6)	10.6	13.8				
		Rotation	BTS	2.4 (1.8)	1.7	3.0	0.084 (–0.09 to 0.32)	0.000	2.6	7.2
			iPi	11.5 (3.8)	10.2	12.8				
		Abduction/Adduction	BTS	11.2 (4.1)	9.8	12.7	0.867 (0.73 to 0.93)	0.711	2.1	6.0
			iPi	11.4 (4.7)	9.8	13.1				
SLS_R (n=34)	THIGH	Flexion/Extension	BTS	43.0 (10.6)	39.3	46.7	0.604 (–0.13 to 0.88)	0.000	3.9	10.7
			iPi	57.1 (9.6)	53.7	60.4				
		Rotation	BTS	13.9 (4.3)	12.4	15.4	0.515 (0.01 to 0.76)	0.891	3.4	9.4
			iPi	14.0 (4.0)	12.6	15.4				
		Abduction/Adduction	BTS	16.1 (7.6)	13.4	18.7	0.758 (0.52 to 0.88)	0.134	4.1	11.4
			iPi	17.6 (5.6)	15.7	19.6				
	SHIN	Flexion/Extension	BTS	26.3 (5.7)	24.3	28.3	0.854 (0.70 to 0.93)	0.051	3.1	8.5
			iPi	27.8 (6.9)	25.4	30.2				
		Rotation	BTS	20.2 (4.4)	18.7	21.8	−0.210 (–0.76 to 0.26)	0.000	4.7	13.0
			iPi	14.1 (4.1)	12.6	15.5				
		Abduction/Adduction	BTS	16.5 (9.3)	13.3	19.7	0.319 (–0.18 to 0.63)	0.000	6.2	17.1
			iPi	10.1 (4.1)	8.7	11.5				
	FOOT	Flexion/Extension	BTS	5.7 (2.7)	4.7	6.6	0.079 (–0.28 to 0.41)	0.000	4.0	11.0
			iPi	11.1 (5.2)	9.3	12.9				
		Rotation	BTS	2.2 (1.9)	1.5	2.9	0.102 (–0.10 to 0.36)	0.000	1.9	5.2
			iPi	8.4 (2.4)	7.6	9.2				
		Abduction/Adduction	BTS	10.2 (2.9)	9.2	11.2	0.707 (0.42 to 0.85)	0.070	2.2	6.0
			iPi	11.2 (3.6)	9.9	12.5				

ICC(2,k), intraclass correlation coefficient (absolute agreement); MDC, minimal detectable change calculated as SEMx1.96x√2. P<0.05; SEM, SE of the measure calculated as the square root of the residual mean square; SLS_L, Single Leg Squat Left; SLS_R, Single Leg Squat Right.

**Table 3:** Range of angles, averaged over the three cycles during the Single Leg Jump test for BTS (considered as reference standard) and IPI software-Kinect configuration

Test	Segment	Movement	System	Mean (SD)	95% CI		ICC(2,k) (95% CI)	P value	SEM	MDC
				(deg)	Lower (deg)	Upper (deg)			(deg)	(deg)
SLJ_L (n=31)	THIGH	Flexion/Extension	BTS	39.0 (9.2)	35.6	42.3	0.491 (–0.17 to 0.82)	0.000	4.4	12.3
			iPi	52.5 (7.9)	49.6	55.4				
		Rotation	BTS	21.6 (5.9)	19.5	23.8	0.622 (0.21 to 0.82)	0.870	4.7	13.0
			iPi	21.8 (6.6)	19.4	24.3				
		Abduction/Adduction	BTS	22.2 (8.6)	19.0	25.3	0.462 (–0.09 to 0.74)	0.216	5.7	15.9
			iPi	20.3 (4.5)	18.7	22.0				
	SHIN	Flexion/Extension	BTS	28.9 (7.5)	26.1	31.6	0.816 (0.62 to 0.91)	0.084	3.6	9.9
			iPi	27.2 (5.4)	25.2	29.2				
		Rotation	BTS	29.0 (4.9)	27.2	30.8	−0.260 (–0.94 to 0.28)	0.000	6.0	16.5
			iPi	22.2 (6.0)	20.0	24.3				
		Abduction/Adduction	BTS	19.2 (4.4)	17.6	20.8	0.529 (–0.20 to 0.84)	0.000	2.2	6.1
			iPi	13.2 (4.0)	11.8	14.7				
	FOOT	Flexion/Extension	BTS	41.6 (10.0)	38.0	45.3	0.487 (–0.13 to 0.82)	0.000	4.2	11.7
			iPi	26.4 (7.9)	23.5	29.3				
		Rotation	BTS	15.0 (4.3)	13.4	16.6	0.461 (–0.04 to 0.73)	0.010	4.3	11.9
			iPi	18.0 (6.1)	15.8	20.2				
		Abduction/Adduction	BTS	23.3 (4.5)	21.6	25.0	0.213 (–0.18 to 0.55)	0.000	3.6	10.1
			iPi	15.1 (4.3)	13.5	16.7				
SLJ_R (n=33)	THIGH	Flexion/Extension	BTS	39.3 (9.1)	36.0	42.5	0.658 (–0.19 to 0.88)	0.000	4.5	12.3
			iPi	47.4 (7.5)	44.7	50.1				
		Rotation	BTS	21.2 (5.3)	19.3	23.1	0.563 (0.15 to 0.78)	0.063	3.8	10.5
			iPi	19.4 (4.5)	17.8	21.0				
		Abduction/Adduction	BTS	17.1 (5.3)	15.2	19.0	0.725 (0.44 to 0.86)	0.036	3.2	8.8
			iPi	15.4 (4.6)	13.8	17.0				
	SHIN	Flexion/Extension	BTS	27.9 (8.9)	24.8	31.1	0.926 (0.84 to 0.96)	0.022	3.0	8.4
			iPi	26.1 (8.3)	23.2	29.1				
		Rotation	BTS	29.2 (5.3)	27.3	31.1	0.297 (–0.21 to 0.63)	0.000	4.1	11.3
			iPi	21.6 (4.9)	19.9	23.4				
		Abduction/Adduction	BTS	16.7 (4.6)	15.1	18.3	0.780 (–0.16 to 0.94)	0.000	1.9	5.2
			iPi	12.8 (5.0)	11.0	14.6				
	FOOT	Flexion/Extension	BTS	43.1 (9.9)	39.6	46.7	0.443 (–0.10 to 0.80)	0.000	4.4	12.1
			iPi	24.5 (9.7)	21.0	27.9				
		Rotation	BTS	14.4 (4.3)	12.9	15.9	0.421 (–0.11 to 0.71)	0.000	3.3	9.2
			iPi	18.0 (4.0)	16.6	19.5				
		Abduction/Adduction	BTS	21.7 (4.9)	19.9	23.4	0.289 (–0.20 to 0.64)	0.000	3.4	9.5
			iPi	13.5 (4.2)	12.0	15.0				

CC(2,k), intraclass correlation coefficient (absolute agreement); MDC, minimal detectable change calculated as SEMx1.96x√2. P<0.05; SEM, SE of the measure calculated as the square root of the residual mean square; SLJ_L, Single Leg Jump Left; SLJ_R, Single Leg Jump Right.

**Table 4:** Range of angles, averaged over the three cycles during the modified counter movement test for BTS (considered as reference standard) and IPI software-Kinect configuration

Test	Segment	Movement	System	Mean (SD)	95% CI		ICC(2,k) (95% CI)	P value	SEM	MDC
				(deg)	Lower (deg)	Upper (deg)			(deg)	(deg)
MCMJ_L (n=33)	THIGH	Flexion/Extension	BTS	59.8 (10.4)	56.1	63.5	0.851 (0.07 to 0.95)	0.000	3.5	9.8
			iPi	65.7 (9.3)	62.4	69.0				
		Rotation	BTS	26.1 (7.3)	23.5	28.7	0.518 (0.00 to 0.77)	0.000	4.7	13.0
			iPi	21.4 (5.1)	19.6	23.2				
		Abduction/Adduction	BTS	26.9 (5.8)	24.8	28.9	0.644 (0.23 to 0.83)	0.002	5.6	15.5
			iPi	31.6 (10.2)	28.0	35.2				
	SHIN	Flexion/Extension	BTS	35.1 (5.8)	33.1	37.2	0.801 (0.60 to 0.90)	0.578	3.4	9.5
			iPi	34.7 (6.1)	32.5	36.8				
		Rotation	BTS	35.7 (9.5)	32.3	39.1	0.571 (0.05 to 0.80)	0.000	5.8	16.1
			iPi	29.7 (6.7)	27.3	32.1				
		Abduction/Adduction	BTS	19.6 (5.2)	17.8	21.4	0.493 (−0.05 to 0.75)	0.000	3.2	8.8
			iPi	16.1 (2.9)	15.1	17.1				
	FOOT	Flexion/Extension	BTS	47.8 (10.8)	44.0	51.7	0.384 (−0.13 to 0.75)	0.000	5.2	14.3
			iPi	28.7 (8.1)	25.9	31.6				
		Rotation	BTS	22.4 (10.4)	18.7	26.1	0.799 (0.59 to 0.90)	0.053	4.8	13.2
			iPi	24.8 (6.0)	22.6	26.9				
		Abduction/Adduction	BTS	23.9 (5.6)	21.9	25.9	0.550 (−0.23 to 0.83)	0.000	2.8	7.7
			iPi	18.3 (3.5)	17.0	19.5				
MCMJ_R (n=34)	THIGH	Flexion/Extension	BTS	56.8 (11.2)	53.0	60.7	0.765 (−0.19 to 0.93)	0.000	3.9	10.9
			iPi	66.3 (10.0)	62.8	69.8				
		Rotation	BTS	23.3 (4.7)	21.7	25.0	−0.280 (-1.55 to 0.36)	0.253	5.8	15.9
			iPi	21.7 (6.1)	19.6	23.8				
		Abduction/Adduction	BTS	28.5 (5.8)	26.4	30.5	0.657 (0.33 to 0.83)	0.059	5.0	13.8
			iPi	26.1 (8.2)	23.2	29.0				
	SHIN	Flexion/Extension	BTS	33.7 (5.2)	31.9	35.5	0.856 (0.71 to 0.93)	0.887	3.0	8.2
			iPi	33.8 (6.4)	31.5	36.0				
		Rotation	BTS	35.4 (6.3)	33.2	37.6	0.049 (−0.50 to 0.45)	0.001	6.0	16.5
			iPi	30.0 (5.8)	28.0	32.1				
		Abduction/Adduction	BTS	21.9 (7.0)	19.5	24.3	−0.030 (−0.50 to 0.36)	0.000	5.4	14.9
			iPi	15.8 (2.9)	14.8	16.8				
	FOOT	Flexion/Extension	BTS	46.0 (8.6)	43.0	49.0	0.202 (−0.13 to 0.55)	0.000	5.9	16.3
			iPi	26.5 (7.1)	24.0	29.0				
		Rotation	BTS	20.9 (4.0)	19.5	22.2	0.277 (−0.22 to 0.60)	0.000	4.5	12.5
			iPi	25.5 (6.1)	23.4	27.6				
		Abduction/Adduction	BTS	25.4 (4.9)	23.7	27.1	0.238 (−0.17 to 0.58)	0.000	4.5	12.5
			iPi	17.1 (3.3)	15.9	18.2				

ICC(2,k), intraclass correlation coefficient (absolute agreement); MCMJ_L, Modified Counter-Movement Jump Left; MCMJ_R, Modified Counter-Movement Jump Right; MDC, minimal detectable change calculated as SEMx1.96x√2. P<0.05; SEM, standard error of the measure calculated as the square root of the residual mean square.

Our results showed excellent between system agreement for shin movement in flexion/extension in all three tests, for both legs. Additionally, during the SLS test excellent agreement was found for thigh and foot adduction/abduction motion.

Results for peak angles are shown in tables 5–7. Between systems agreement was excellent for knee flexion in all tests for both legs.

**Table 5:** Peak angles averaged over the three cycles during the Single Leg Squat test for BTS (considered as reference standard) and IPI software-Kinect configuration

Test	Movement	System	Mean (SD)	95% CI		ICC(2,k) (95% CI)	P value	SEM	MDC
			(deg)	Lower (deg)	Upper (deg)			(deg)	(deg)
SLS_L (n=34)	Hip flexion	BTS	−30.3 (17.5)	−36.4	−24.2	0.896 (–0.09 to 0.97)	0.000	4.1	11.3
		iPi	−40.6 (18.0)	−46.9	−34.3				
	Hip adduction	BTS	−18.6 (5.5)	−20.6	−16.7	0.749 (0.49 to 0.88)	0.029	3.3	9.2
		iPi	−20.5 (5.4)	−22.4	−18.6				
	Knee flexion	BTS	30.0 (8.6)	27.0	33.0	0.932 (0.71 to 0.98)	0.000	2.5	6.8
		iPi	32.8 (8.5)	29.8	35.7				
	Knee adduction	BTS	−17.5 (6.1)	−19.6	−15.3	0.830 (0.57 to 0.92)	0.001	2.6	7.3
		iPi	−15.3 (4.5)	−16.8	−13.7				
SLS_R (n=34)	Hip flexion	BTS	−49.1 (14.8)	−54.3	−44.0	0.767 (–0.12 to 0.94)	0.000	3.9	10.9
		iPi	−63.9 (14.7)	−69.0	−58.7				
	Hip adduction	BTS	22.8 (4.7)	21.1	24.4	0.684 (–0.13 to 0.89)	0.000	2.6	7.2
		iPi	27.1 (5.1)	25.3	28.9				
	Knee flexion	BTS	25.9 (9.8)	22.5	29.4	0.947 (0.83 to 0.98)	0.001	2.8	7.8
		iPi	28.5 (10.7)	24.8	32.3				
	Knee adduction	BTS	18.3 (5.8)	16.3	20.3	0.665 (0.08 to 0.86)	0.000	3.0	8.2
		iPi	14.7 (3.8)	13.4	16.1				

ICC(2,k), intraclass correlation coefficient (absolute agreement); MDC, minimal detectable change calculated as SEMx1.96x√2. P<0.05; SEM, SE of the measure calculated as the square root of the residual mean square; SLS_L, Single Leg Squat Left; SLS_R, Single Leg Squat Right.

**Table 6:** Peak angles averaged over the three cycles during the Single Leg Jump test for BTS (considered the gold standard) and IPI software-Kinect configuration

Test	Movement	System	Mean (SD)	95% CI		ICC(2,k) (95% CI)	P value	SEM	MDC
			(deg)	Lower (deg)	Upper (deg)			(deg)	(deg)
SLJ_L (n=31)	Hip flexion	BTS	−27.1 (16.4)	−33.2	−21.1	0.890 (−0.05 to 0.97)	0.000	4.3	12.0
		iPi	−36.6 (16.6)	−42.7	−30.5				
	Hip adduction	BTS	−19.0 (5.5)	−21.0	−17.0	0.826 (0.64 to 0.92)	0.744	2.9	7.9
		iPi	−18.7 (4.9)	−20.5	−16.9				
	Knee flexion	BTS	31.4 (10.6)	27.5	35.3	0.951 (0.90 to 0.98)	0.694	3.0	8.3
		iPi	31.7 (8.7)	28.5	34.9				
	Knee adduction	BTS	−16.5 (4.9)	−18.3	−14.7	0.883 (0.32 to 0.96)	0.000	1.7	4.7
		iPi	−14.0 (5.0)	−15.9	−12.2				
SLJ_R (n=33)	Hip flexion	BTS	−46.8 (11.8)	−51.0	−42.6	0.649 (−0.19 to 0.89)	0.000	5.0	14.0
		iPi	−60.7 (11.6)	−64.8	−56.6				
	Hip adduction	BTS	23.1 (5.5)	21.2	25.0	0.901 (−0.04 to 0.97)	0.002	3.7	10.2
		iPi	26.1 (5.9)	24.0	28.2				
	Knee flexion	BTS	27.5 (11.2)	23.6	31.5	0.956 (0.91 to 0.98)	0.513	3.1	8.6
		iPi	28.1 (9.9)	24.5	31.6				
	Knee adduction	BTS	18.8 (4.9)	17.0	20.5	0.726 (–0.21 to 0.92)	0.000	2.0	5.5
		iPi	14.3 (4.5)	12.6	15.9				

ICC(2,k), intraclass correlation coefficient (absolute agreement); MDC, minimal detectable change calculated as SEMx1.96x√2. P<0.05; SEM, SE of the measure calculated as the square root of the residual mean square; SLJ_L, Single Leg Jump Left; SLJ_R, Single Leg Jump Right.

**Table 7:** Peak angles averaged over the three cycles during the modified counter movement test for BTS (considered the gold standard) and IPI software-Kinect configuration

Test	Movement	System	Mean (SD)	95% CI		ICC(2,k) (95% CI)	P value	SEM	MDC
			(deg)	Lower (deg)	Upper (deg)			(deg)	(deg)
MCMJ_L (n=33)	Hip flexion	BTS	−49.4 (18.7)	−56.0	−42.8	0.947 (0.33 to 0.99)	0.000	3.7	10.2
		iPi	−56.3 (18.4)	−62.8	−49.7				
	Hip adduction	BTS	−18.8 (6.0)	−20.9	−16.7	0.792 (0.17 to 0.92)	0.000	2.5	7.0
		iPi	−15.3 (4.8)	−17.1	−13.6				
	Knee flexion	BTS	35.8 (10.1)	32.2	39.4	0.954 (0.80 to 0.98)	0.000	2.4	6.6
		iPi	38.4 (9.7)	35.0	41.9				
	Knee adduction	BTS	−16.3 (6.0)	−18.5	−14.2	0.873 (0.59 to 0.95)	0.000	2.4	6.5
		iPi	−13.9 (5.6)	−15.9	−12.0				
MCMJ_R (n=34)	Hip flexion	BTS	−65.7 (14.5)	−70.8	−60.7	0.846 (–0.14 to 0.96)	0.000	3.5	9.7
		iPi	−76.4 (13.9)	−81.3	−71.6				
	Hip adduction	BTS	24.0 (5.9)	21.9	26.0	0.713 (0.12 to 0.88)	0.000	3.1	8.5
		iPi	20.1 (5.0)	18.4	21.9				
	Knee flexion	BTS	31.0 (8.3)	28.1	33.9	0.945 (0.81 to 0.98)	0.000	2.5	6.8
		iPi	33.4 (9.6)	30.1	36.8				
	Knee adduction	BTS	16.8 (5.2)	15.0	18.6	0.742 (–0.01 to 0.91)	0.000	2.5	6.9
		iPi	13.1 (4.8)	11.4	14.7				

ICC(2,k), intraclass correlation coefficient (absolute agreement); MCMJ_L, Modified Counter-Movement Jump Left; MCMJ_R, Modified Counter-Movement Jump Right; MDC, minimal detectable change calculated as SEMx1.96x√2. P<0.05; SEM, SE of the measure calculated as the square root of the residual mean square.

Biases and limits of agreement (table 8) (online supplementary material 1: Bland-Altman plots) were documented. The mean differences are relatively low especially for hip adduction and knee flexion and adduction. For most of the measures examined, no systematic error is detected. For hip flexion, however, there appears to be a systematic error of approximately 10°.

10.1136/bmjsem-2018-000441.supp1 Supplementary data

**Table 8:** 95% LOA and the bias of the motion capture systems

Test	Movement	Lower LOA (deg)	Upper LOA (deg)	Bias (deg)
SLS_L	Hip flexion	−0.9	21.6	10.3
	Hip adduction	−7.4	11.1	1.8
	Knee flexion	−9.6	4.1	−2.8
	Knee adduction	−9.5	5.0	−2.2
SLS_R	Hip flexion	3.8	25.7	14.7
	Hip adduction	−11.6	2.9	−4.4
	Knee flexion	−10.4	5.2	−2.6
	Knee adduction	−4.7	11.8	3.6
SLJ_L	Hip flexion	−2.5	21.4	9.5
	Hip adduction	−8.2	7.7	−0.2
	Knee flexion	−8.6	8.0	−0.3
	Knee adduction	−7.1	2.2	−2.5
SLJ_R	Hip flexion	−0.1	27.8	13.9
	Hip adduction	−13.2	7.2	−3.0
	Knee flexion	−9.1	8.1	−0.5
	Knee adduction	−0.9	10.0	4.5
MCMJ_L	Hip flexion	−3.3	17.1	6.9
	Hip adduction	−10.4	3.6	−3.4
	Knee flexion	−9.2	4.0	−2.6
	Knee adduction	−8.9	4.1	−2.4
MCMJ_R	Hip flexion	1.0	20.4	10.7
	Hip adduction	−4.7	12.4	3.8
	Knee flexion	−9.2	4.4	−2.4
	Knee adduction	−3.1	10.6	3.8

LOA, limits of agreement; MCMJ_L, Modified Counter-Movement Jump Left; MCMJ_R, Modified Counter-Movement Jump Right; SLS_L, Single Leg Squat Left; SLS_R, Single Leg Squat Right.

## Discussion

Here, we have established, for the first time, validity values for SLS, CMJ and MCMJ in a cohort of professional athletes using a 2 camera markerless motion capture system (Kinect v2).

Our results indicate that a dual Kinect v2 configuration is a valid tool for assessment of sagittal plane knee range and peak angles, during squat and jumping tests. Additionally, during the SLS test excellent agreement between systems was found for thigh and foot adduction/abduction motion.

Although agreement improved when using two cameras configuration instead of one,^27^ the between system agreement varied widely, especially for movements of clinical interest like hip flexion, hip adduction and knee adduction. There was also variability in agreement for different joints and different parameters. For example, shin ab/adduction showed better reliability and validity when considering the peak values in comparison to the results from individual tests. Clinical interpretation is therefore recommended for each approach (eg, individual trials vs averaged values, vs peak values). It may be argued that, in the context of risk of an acute anterior cruciate ligament injury, the peak shin adduction is a more important metric than the average across a number of trials whereas in ‘overuse’ type injuries average values may be a more sensible estimator Also, poor agreement was found regarding all rotational movements. Regarding peak angles, we noticed slightly, but inconsistently, better results found for left side compared with right. Positioning one camera on the left side of the athletes may have influenced these results. Importantly, the estimation of the MDC allows better interpretation of the individual kinematic parameters of interest for future studies and allows for adequate planning (power analyses) of intervention trials. For example, it is suggested that knee abduction at initial contact and peak during a drop jump task is predictive of subsequent ACL injury—the between group differences being 8.4° and 7.6°, respectively, for those who were subsequently injured and those who were not.^28^ We suggest that this infers the amount of variability between trials for a given subject is likely so large as to exceed these suggested cut-points. In comparison, studies examining changes in hip and knee peak flexion during a landing task, after a fatigue protocol, report 5.1⁰ hip flexion and 6.7⁰ knee flexion^29^ and 7⁰ increase in peak knee flexion before and after a general fatigue protocol.^30^ In comparison to the displayed MDC values here, we suggest that both the markerless and marker-based approaches can readily detect such changes. Further to this, it was noted that the hip flexion angle appeared to have a systematic error of approximately 10° when comparing the markerless and marker-based systems. Post processing (ie, subtracting 10° from all measures) could simply remove this artefact and result in more accurate measures. It is uncertain from where this shift arises; however, the closed nature of the processing conducted through the markerless software capture and subsequent processing likely render this a difficult problem to resolve.

Recent studies using Kinect v2 multiple cameras^19 20^ found excellent between systems agreement when measuring spatiotemporal gait parameters. It should be noted, however, that these studies examined gait, not higher speed movements examined here.

The advantages of the Kinect approach were the much shorter set-up time and much lower (financial) cost of the equipment. Processing time was approximately 7 min for each test and the results derived are for the whole body. We suggest that consideration of the accuracy presented here along with these advantages will allow clinicians to better assess if this approach would be viable for their specific situation.

Some limitations should be considered in the interpretation of the results of this study. Differences in the definition of reference systems and processing between the two systems may have an important role in the extracted results. Additionally, position chosen for markerless cameras may have had a negative effect on the results obtained. Variations in footwear type and sole height may have caused variations in ankle joint centre detection, reducing measurement accuracy. Additionally, markers on the footwear may have affected results for the foot—potentially this is a source of the movement differences detected by Kinect and the BTS system. Importantly, Microsoft recently discontinued production of the Kinect v2 camera. Although the devices remain available for purchase online and in physical retail stores at the time of writing, this will change in the future.

Future studies are recommended to test the clinical utility of Kinect v2–iPi software configuration using more than two cameras. Cameras set up at 45° from the frontal plane may positively influence the extracted data. Future investigations should use standardised footwear or barefoot conditions to improve ankle visualisation and improve measurement of ankle joint kinematics. In conclusion, this study supports the use of dual Kinect v2 configuration with the iPi software as a valid tool for assessment of sagittal knee kinematic parameters in athletes.
