# Supplementary material for: Dual Kinect v2 system can capture lower limb kinematics reasonably well in a clinical setting: concurrent validity of a dual camera markerless motion capture system in professional football players
Source: BMJ Open Sport Exerc Med. 2018 Dec 17;4(1):e000441. doi: 10.1136/bmjsem-2018-000441 (PMC6307561; doi:10.1136/bmjsem-2018-000441)
Supplement: Supplementary data [file bmjsem-2018-000441supp001.docx]

**SUPPLEMENTARY MATERIAL**


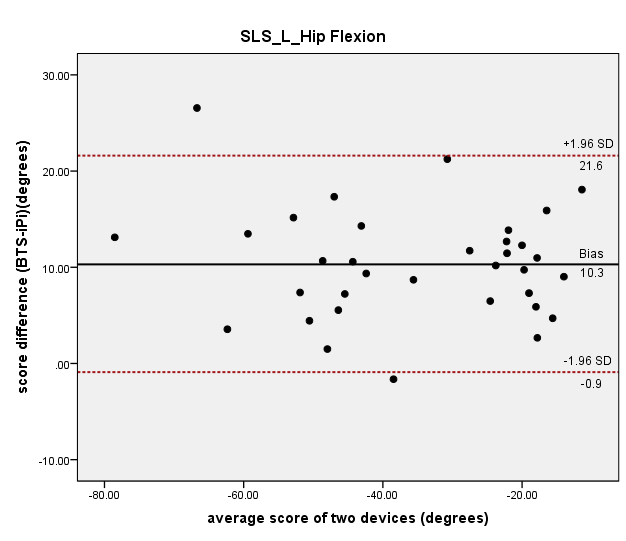

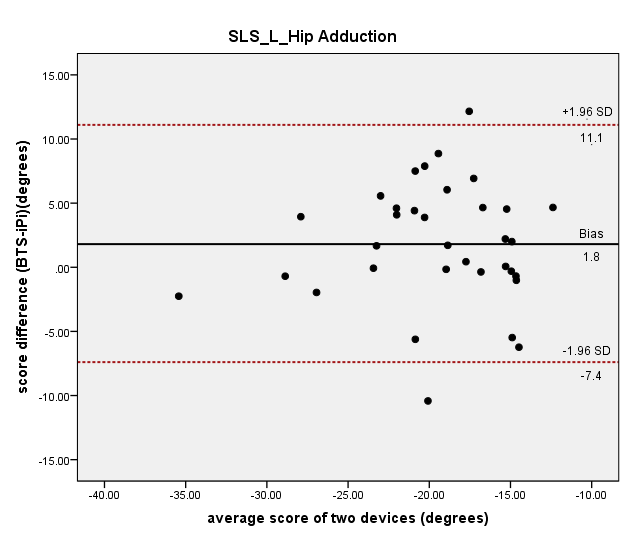


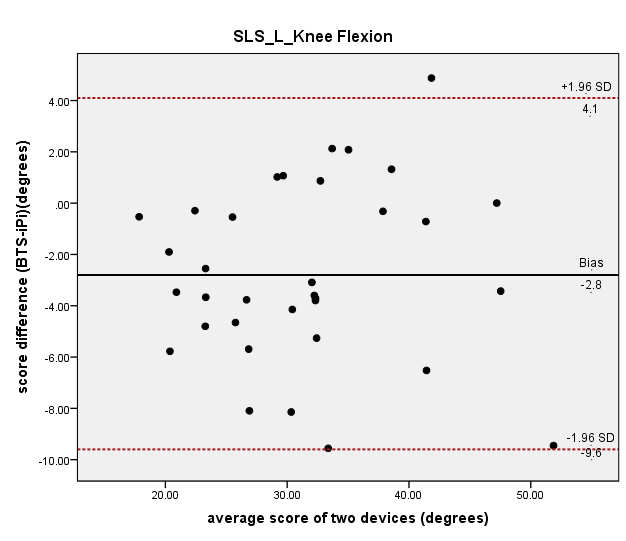

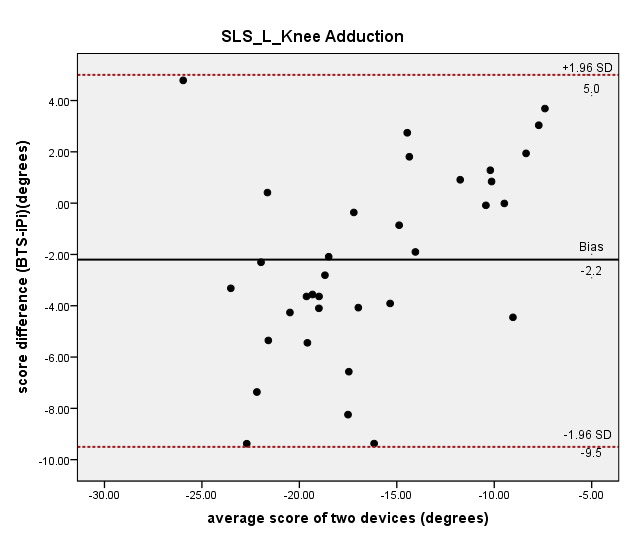


**Figure 4.7 Bland and Altman plots for Single Leg Squat test for Left leg.**


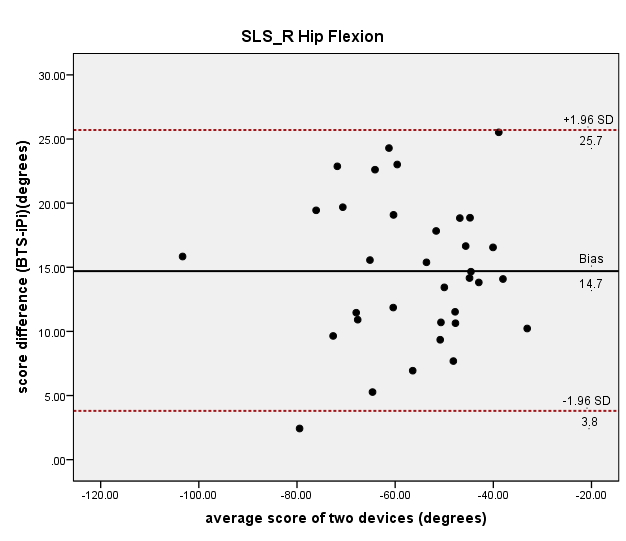

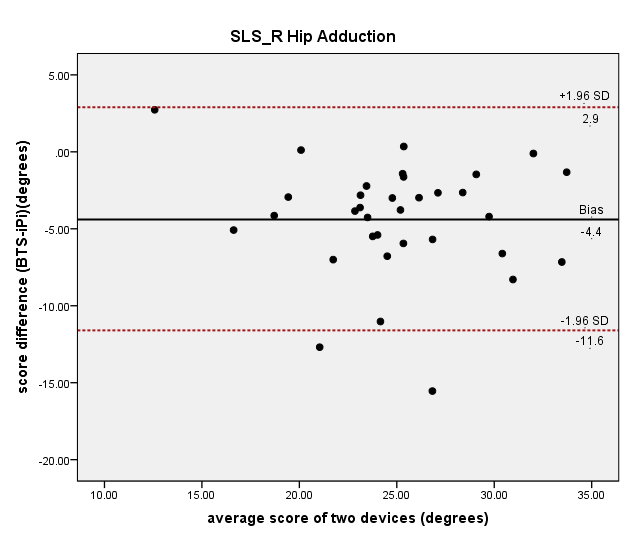


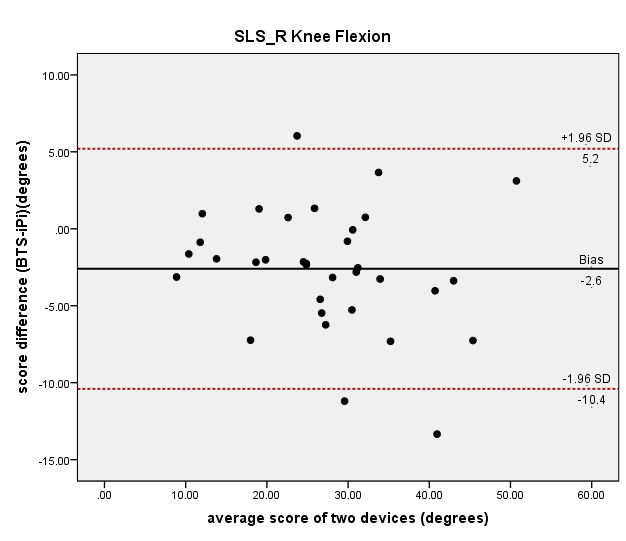

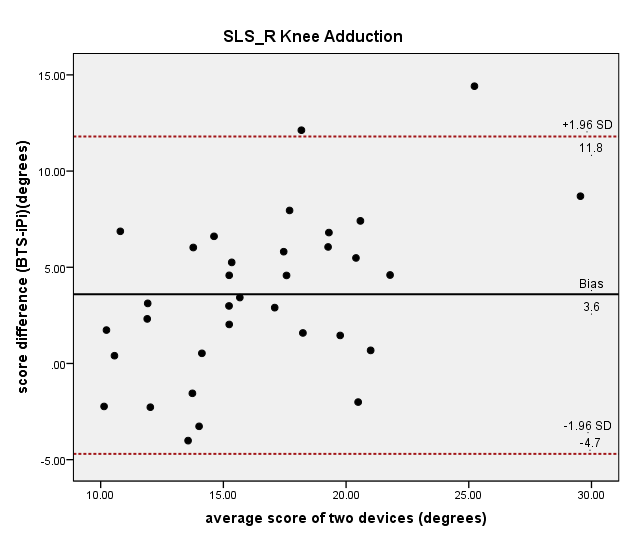


**Figure 4.8 Bland and Altman plots for Single Leg Squat test for Right leg.**


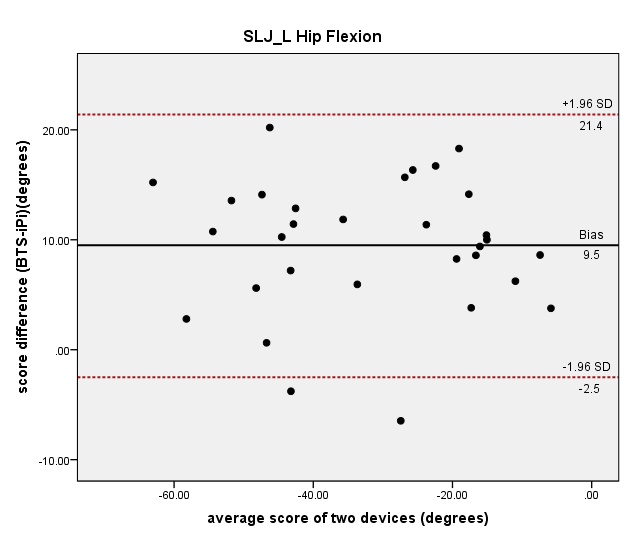

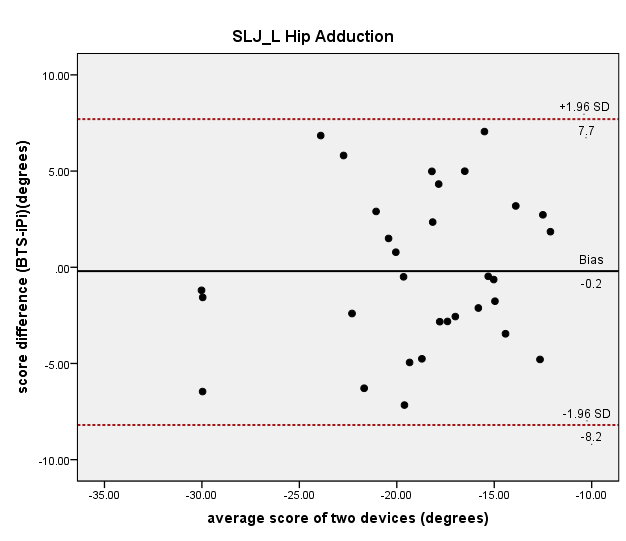


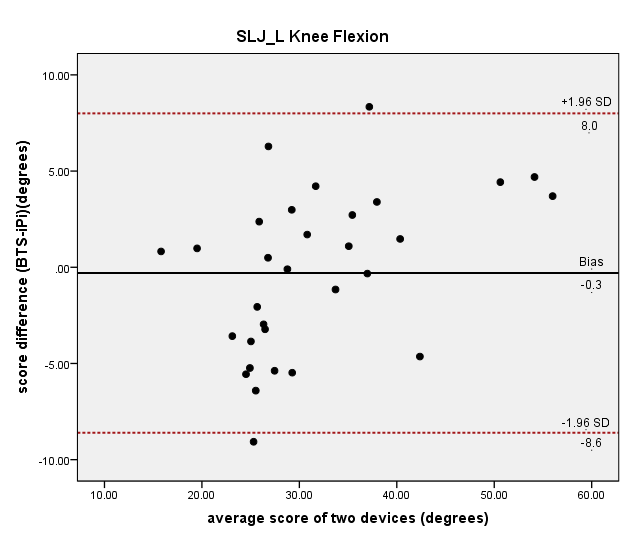

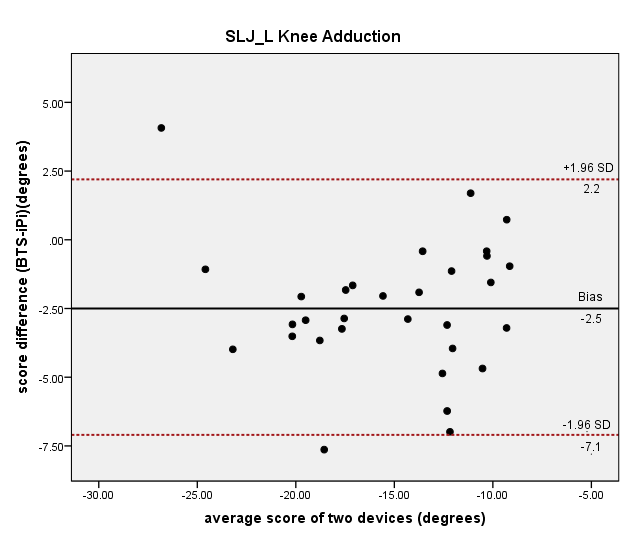


**Figure 4.9 Bland and Altman plots for Single Leg Jump test for Left leg.**


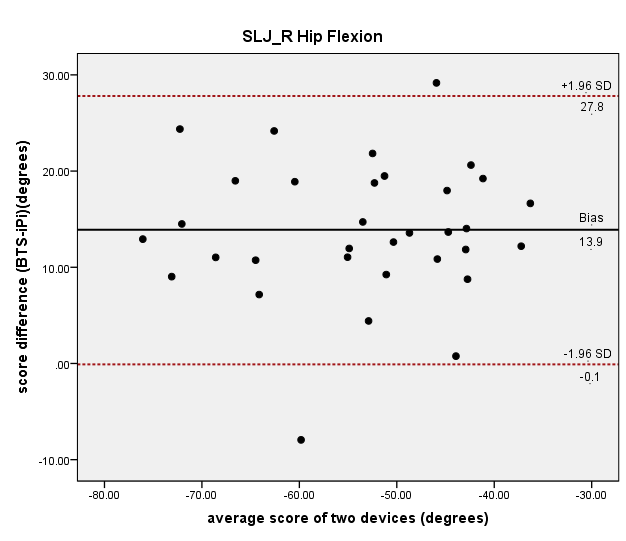

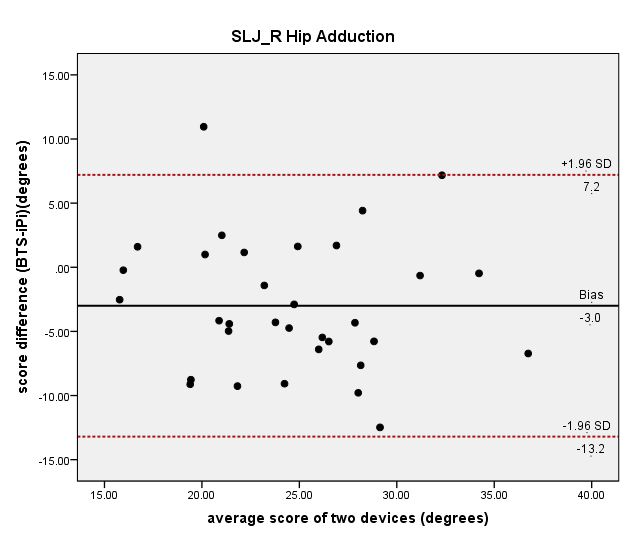


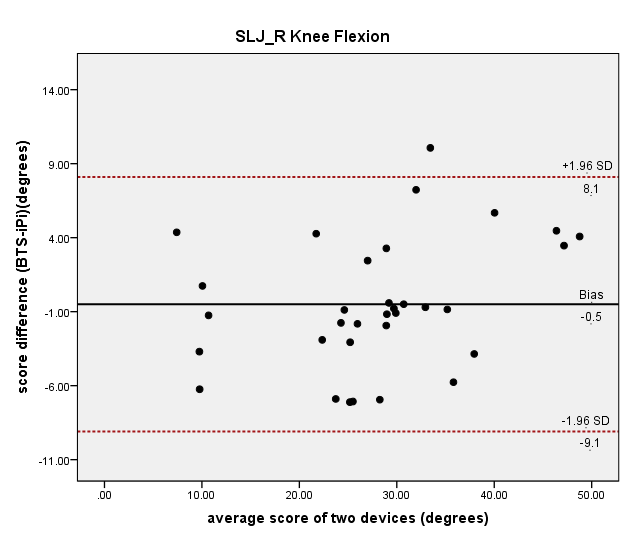

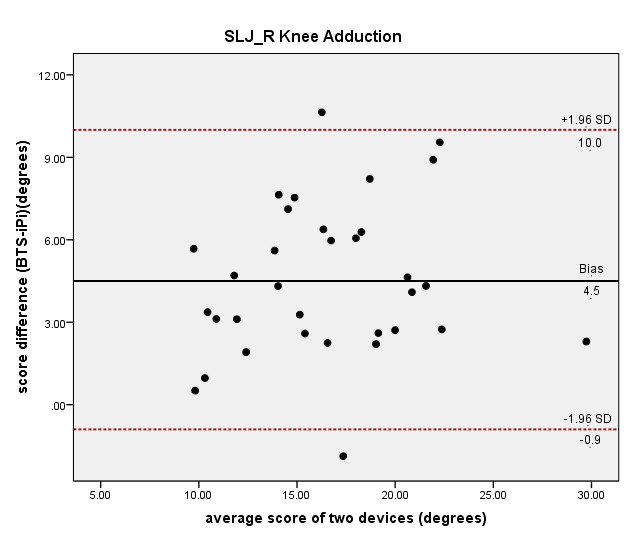


**Figure 4.10 Bland and Altman plots for Single Leg Jump test for Right leg.**


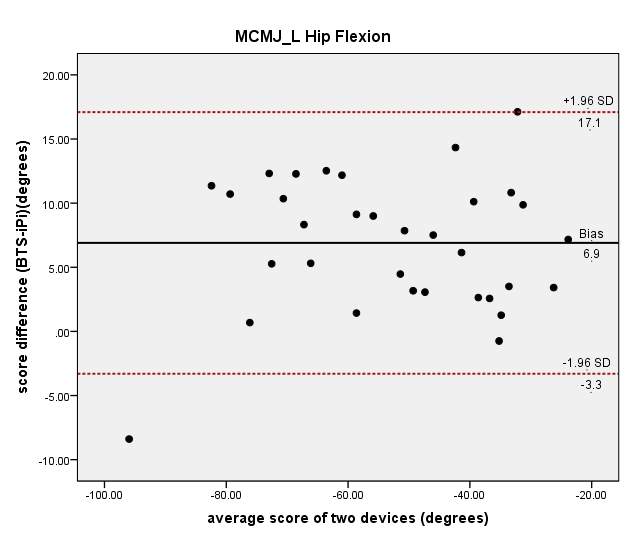

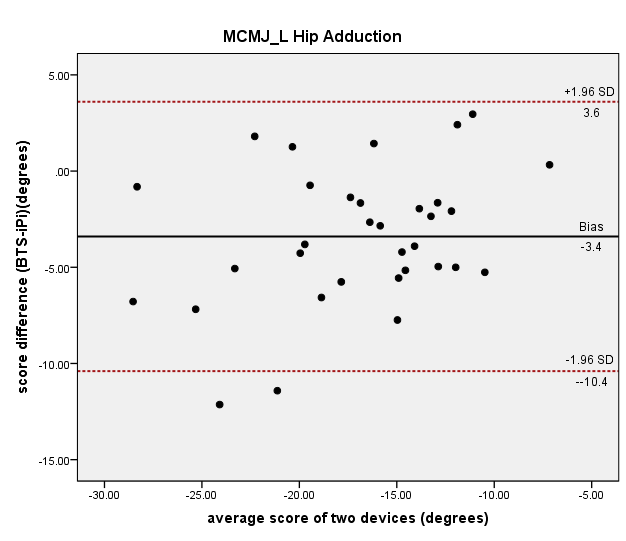


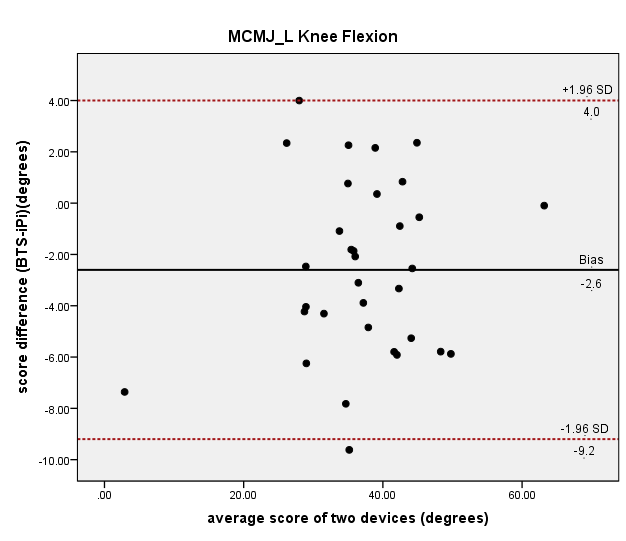

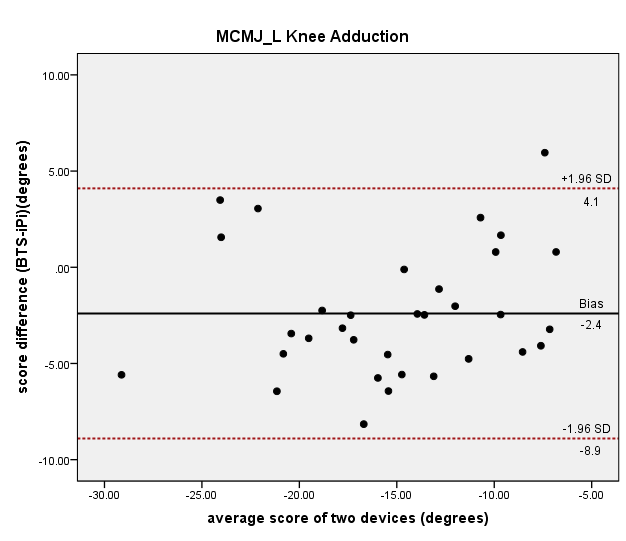


**Figure 4.11 Bland and Altman plots for Modified Counter-Movement Jump test for Left leg.**


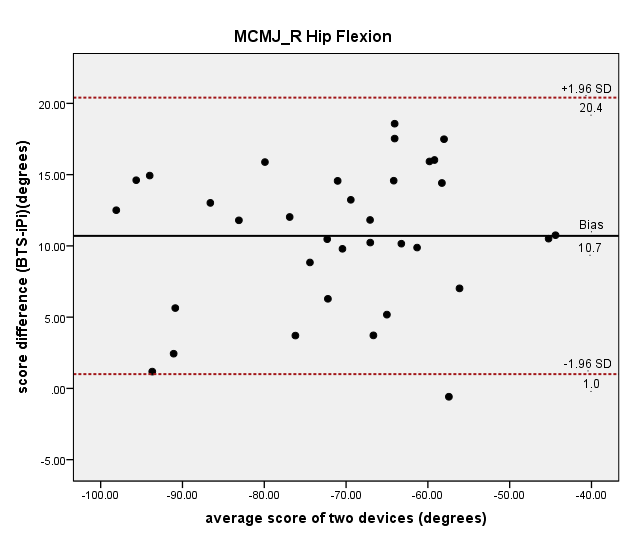

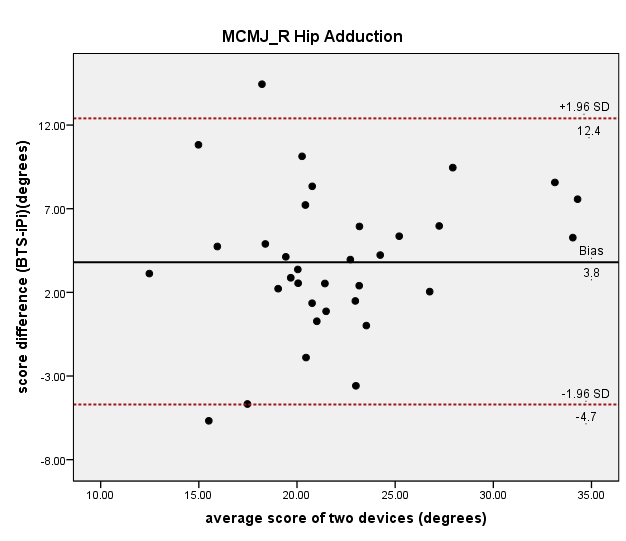


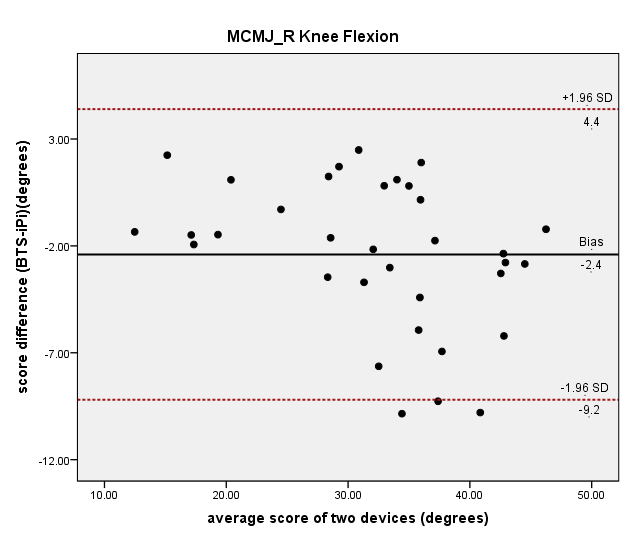

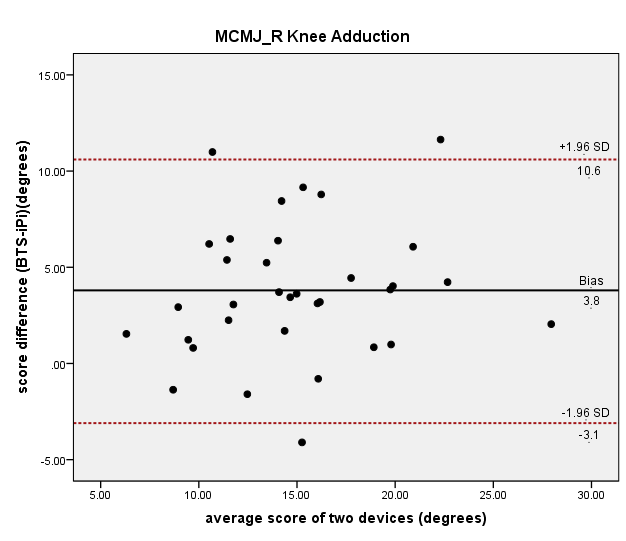


**Figure 4.12 Bland and Altman plots for Modified Counter-Movement Jump test for Right leg.**
